# Nucleus Reuniens-Elicited Delta Oscillations Disable the Prefrontal Cortex in Schizophrenia

**DOI:** 10.3390/cells14191545

**Published:** 2025-10-03

**Authors:** Robert P. Vertes, Stephanie B. Linley

**Affiliations:** 1 Center for Complex Systems and Brain Sciences, Florida Atlantic University, Boca Raton, FL 33431, USA; 2 Department of Psychological Sciences, University of Northern Colorado, Greeley, CO 80639, USA; stephanie.linley@unco.edu

**Keywords:** glutamate, dopamine, thalamus, hippocampus, reticular thalamic nucleus, cognition, memory

## Abstract

Schizophrenia (SZ) is a severe mental disorder associated with an array of symptoms characterized as positive, negative and cognitive dysfunctions. While SZ is a multifaceted disorder affecting several regions of the brain, altered thalamocortical systems have emerged as a leading contributor to SZ. Specifically, it has been shown that: (1) the thalamus is functionally disconnected from the prefrontal cortex (PFC) in SZ; (2) neural activity and blood flow to the PFC are greatly diminished in SZ (hypofrontality); and (3) delta oscillations are abnormally present in the PFC during the waking state in SZ. We suggest that the abnormal delta oscillations drive the other PFC signs of SZ. Specifically, decreases in energy required to maintain delta, would initiate the reduced PFC perfusion of SZ (hypofrontality), and contribute to the ‘mismatched’ thalamic and PFC activity of SZ. As SZ involves glutamate (NMDAR) hypofunction and dopamine hyperfunction, both NMDAR antagonists and dopamine agonists produce marked increases in delta oscillations in nucleus reuniens (RE) of the thalamus and its target structures, including the PFC. This would suggest that RE is a primary source for the elicitation of PFC delta activity, and the presence of delta during waking (together with associated signs) would indicate that the prefrontal cortex is disabled (or non-functional) in schizophrenia.

## 1. Introduction

Schizophrenia (SZ) is a severe mental disorder associated with an array of symptoms that have been characterized as positive (delusions, hallucinations), negative (flat affect, anhedonia) and cognitive dysfunctions (altered working memory and executive functions) [1]. While antipsychotics are generally effective for treating the positive symptoms of SZ, they are largely ineffective for treating the negative symptoms and the cognitive deficits of SZ. Treatment-resistant cognitive impairments of SZ represent a major public health issue and the mechanisms underlying the cognitive dysfunctions of SZ are poorly understood [1].

## 2. Thalamocortical (TC) Functional Dysconnection in Schizophrenia (SZ)

It is widely held that SZ is a developmental disorder, in large part, involving disruptions of thalamocortical (TC) systems [2,3,4,5,6,7,8,9,10,11,12,13,14,15,16,17]. Several resting state or task-based functional MRI studies have reported that the thalamus is ‘disconnected’ from the cortex in SZ. Specifically, there is a marked reduction in thalamic connections with the cortex, mainly with the prefrontal cortex (PFC) in SZ (hypoconnectivity), whereas there is a parallel increase in thalamic connections with sensory/motor regions of the cortex (hyperconnectivity) [2,4,5,7,9,15,16,17,18,19]. In this regard, Giraldo-Chica and Woodward [11] stated that “thalamocortical functional dysconnectivity is a core neurobiological abnormality in SZ”.

TC dysconnectivity has been demonstrated in early stage and chronic SZ as well as in individuals at high risk for SZ [4,5,17,20]. It has further been reported [21] that healthy siblings of SZ patients, who share half of their siblings’ genotype and are at much greater risk for developing SZ, also exhibited decreased thalamic connectivity with the PFC—but to a lesser degree than for SZ patients. Interestingly, however, the healthy siblings did not show the SZ-associated increase in TC connectivity with sensorimotor cortices suggesting that this emerges with the development of the disease [21]. To this point, Ramsay [22] showed that SZ *patients* displaying pronounced TC hypoconnectivity also exhibited TC sensorimotor hyperconnectivity suggesting both abnormalities develop with disease progression and may arise from a common mechanism.

The degree to which the thalamus and cortex are disconnected in SZ appears to directly correspond to the severity of the disorder [23]. Szeszko et al. [24] compared TC connectivity of subjects with schizotypal personality disorder (PSD) to healthy controls (HC) and SZ patients and reported that TC connectivity was generally comparable between PSD and HC subjects but significantly reduced for SZ patients. Interestingly for PSD patients stronger functional connections (FC) were associated with less severe symptoms leading the authors to suggest that strong TC connections may protect PSD patients from more serious psychotic symptoms.

Regarding the relationship between TC dysconnections and symptoms, Ramsay et al. [25] described psychopathological changes, including alterations of cognition, associated with TC dysconnections of SZ, bipolar (BP) disorders and first-degree relatives of SZ/BP patients. They reported [25] that positive and negative symptoms as well as cognitive impairments were directly correlated with reduced TC connectivity for SZ and BP patients—but to a much lesser degree for first-degree relatives. Wu et al. [26], using resting state and task-based fMRI techniques, confirmed TC hypoconnectivity and hyperconnectivity with the PFC and sensory cortical regions, respectively, of SZ patients, and further reported that reductions in thalamo-PFC connections produced significant working memory (WM) deficits. These findings suggest that the well-documented cognitive impairments of SZ [27,28,29,30,31,32] may mainly involve a functional dysconnection of the thalamus with the PFC.

It has been proposed [10,33] that thalamo-PFC dysfunctions result from a disturbance of normal brain development in the transition from adolescence to adulthood—a period of pronounced developmental changes in the functional connectivity of the brain. In this regard, Berge et al. [34] examined the progression of TC connectivity over a 12-month period from the onset of SZ and showed that the initial reduction in thalamo-PFC connectivity remained stable over the entire period—thereby concluding that these functional changes occur relatively early in the development of SZ and only minimally decline thereafter.

## 3. The 22q11.2 Deletion Syndrome for SZ—Concentrating on TC Functional Connections

The deletion of chromosome 22q11.2 is one of the strongest genetic risk factors for SZ; that is, the syndrome occurs in approximately 1 to 3000 to 6000 live births, typically involves the deletion of ~46 protein-coding genes, and approximately 25% of carriers of the deletion develop SZ [35,36]. As reviewed above, a hallmark of SZ is TC dysconnectivity, including heightened thalamic connections with sensorimotor cortices. In this regard, Chun and associates [37,38], using the mouse model for the 22q11.2 deletion syndrome (22q11DS mice), showed that thalamic projections to the auditory cortex (AUD) were disrupted in the mice which led to alterations of the acoustic startle response—comparable to that of SZ patients. The disrupted thalamo-AUD connections were associated with an abnormal elevation of dopamine D2 receptors in the thalamus, and this, in turn, rendered the thalamus acutely sensitive to various antipsychotics which partially reversed the effects of the altered TC circuity of the 22q11DS mice. The disruption of this TC circuity in the mice typically developed at about four months of age, which corresponds to young adulthood in humans [38].

Consistent with findings in the mouse model, Schleifer and co-workers [39] examined patterns of TC connectivity in young subjects (7–26 years of age) with the 22q11.2 syndrome and described reduced connectivity with frontoparietal cortices and enhanced thalamic connections with sensorimotor cortices—similar to patterns shown for classic SZ. In a like manner, Mancini et al. [40] described developmental changes in thalamic nuclei and their functional connections with the cortex of subjects, aged 8–35 years, with the 22q11.2 deletion syndrome (22qdel) who experienced auditory hallucinations (AHs). Among the findings, they showed [40] that the volume of the medial geniculate nucleus (MGN) was bilaterally reduced in 22qdel subjects with AHs, beginning in late childhood with a progressive loss to adulthood. This was ‘paradoxically’ associated with (or coupled to) enhanced FCs of the MGN with the auditory cortex (AUD) and with speech processing regions of the cortex. They tentatively suggested that the MGN-AUD hyperconnectivity may be a compensatory response to the loss of volume of the MGN.

In a follow-up study [41] decreases in the volume of auditory structures of 22q11.2 subjects interestingly did not lead to a reduced amplitude of the mismatch negativity response (MMN)—as typically found in SZ. They suggested that alterations of the MMN may involve disrupted MGN-AUD functional connections rather than absolute changes in the volume of the MGN. Finally, they reported [41] that the “only other” thalamic nucleus (aside from the MGN) which showed morphological changes (reduced volume) in 22qdel subjects with AHs was the nucleus reuniens (RE) of the midline thalamus. Additionally, the decrease in volume in RE was correlated with a reduced volume of CA1 of the hippocampus (HF) for these subjects [40,42]. As the RE and CA1/HF are reciprocally connected [43,44,45,46,47,48], this raised the possibility that alterations of RE, and its connections with the HF, may contribute to the cognitive dysfunctions of 22qdel patients. Supporting this, Latreche et al. [49] described deficits in verbal learning linked to reductions in the volume of the hippocampus in 22q.11.2DS subjects.

## 4. Thalamocortical Dysconnection in Sleep: Parallels to TC Dysconnectivity in SZ

As is well established, there is a dramatic shift in the patterns of cortical EEG activity from wakefulness to sleep—which is dependent on thalamic actions on the cortex [50,51]. In brief, reductions in ascending excitatory inputs to cells of the reticular nucleus and midline/medial nuclei of the thalamus hyperpolarizes them leading to a de-inactivation of the low threshold Ca^2+^ channels which upon rebound depolarization, the cells fire in bursts at low frequencies giving rise to slow wave (delta) activity in the cortex [50,51].

Relatively few studies have compared thalamocortical functional connectivity (FC) in sleep and waking states. In an early report, using the central medial nucleus (CM) of the thalamus as a seed, Picchioni et al. [52] described a progressive decrease in the FC of the CM with the frontal cortex from waking to non-REM (NREM) sleep. They suggested that this TC dysconnection in sleep was equivalent to a complete functional deafferentation of the PFC during sleep [52]. In like manner, Hale et al. [53] described alterations of FC of thalamic subnuclei with cortical targets specifically demonstrating decreases in TC connections with frontal cortices but interestingly, increases in thalamic connections with sensorimotor cortices during NREM sleep. This pattern of TC connections during NREM sleep directly parallels that shown for SZ.

Comparable to NREM sleep, there appears to be a similar loss of TC connectivity in REM sleep—or specifically during select periods of REM sleep in which ‘delta’ activity is present in thalamic nuclei, mainly in the medial pulvinar [54,55,56]. For instance, Bastuji et al. [56] demonstrated that during periods of REM sleep characterized by *thalamic* delta rhythms (>50% of REM sleep) there was a dramatic decrease in thalamocortical and cortico-cortical FC relative to the waking state. The authors [56] suggested that the TC dysconnectivity during (thalamic) delta activity in REM sleep may largely be responsible for the bizarre properties of dreams (hallucinations, delusions) and speculated that TC dysconnections in certain neurological disorders (e.g., SZ) could similarly account for the altered sensory and cognitive processes of those conditions.

## 5. The Prefrontal Cortex (PFC) Is Disabled in SZ: PFC Delta Activity and Hypofrontality in SZ

### 5.1. PFC Delta Oscillations in SZ

Seemingly in line with the loss of functional connections of the thalamus with the cortex/PFC in SZ, several reports, beginning early on, described the abnormal presence of delta activity in the *waking state* in SZ [57,58,59,60,61,62,63,64,65,66,67]. As discussed below, this includes first-degree relatives of SZ patients, individuals at high risk for SZ, the early (non-medicated) stage of SZ, and chronic SZ.

In an early report, Sponheim et al. [57], analyzing cortical EEG activity of SZ and control subjects described significantly increased delta (1–3 Hz) and theta (3–8 Hz) oscillations and reduced alpha and beta activity for SZ patients during waking. Significantly, the changes were the same for first episode and chronic SZ—indicating that effects did not involve medications or SZ duration. In a series of studies using magnetoencephalography (MEG), Fehr and associates [60,61,63] similarly reported an increase in slow wave activity (delta and theta) during waking in SZ patients, and further showed, using source density analysis, that effects were predominantly localized to frontal and temporal regions of the cortex. They remarked that, aside from sleep, delta activity is typically associated with brain damage or coma so its presence during waking of SZ patients would likely signify severe alterations of normal cortical activity.

In a meta-analysis examining EEG profiles of SZ patients, Boutros et al. [65] assessed 53 articles and, of these, 15 met the criteria of accurately comparing SZ subjects to controls. Of the 15 articles, virtually all described a similar pattern of cortical EEG activity during waking: increases in delta and theta power and decreases in alpha power in SZ. Accordingly, they proposed that this EEG profile “emerges as a strong candidate for development into a diagnostic test” for SZ patients. In an examination of recent onset, drug naive SZ patients compared to controls, John et al. [68] confirmed previous findings of significantly enhanced delta activity during waking in SZ—which was associated with negative symptoms of SZ. In contrast, however, to some earlier reports, they described *decreases* rather than increases in theta activity for SZ subjects and suggested [68] that previously reported increases in theta in SZ may have been the result of neuroleptic treatment. Supporting this, decreases in theta power were found with the onset of SZ but reversed to increases following neuroleptic administration [69].

With some exceptions [62,70], several additional studies confirm the earlier demonstration [68] that delta oscillations of waking are correlated with negative symptoms of SZ [58,61,71,72,73,74,75,76,77]. This includes subjects at high risk (or ultra-high risk) for SZ [78] who transitioned to psychosis [79]. For instance, Chen et al. [80] reported that (PFC) delta activity was associated with negative symptoms (and cognitive deficits) of SZ patients and thus concluded that “low frequency oscillatory abnormalities appear to be the primary abnormality in schizophrenia at rest as well as during tasks”. Finally, Sponheim et al. [81] described a general slowing of cortical EEG activity in SZ patients and suggested, in effect, that the cortical slowing in SZ could contribute to the TC ‘mismatch’ of SZ—or serve as the “basis for the functional dysconnectivity of the disorder”.

### 5.2. Frontal Cortical (FrC) Hypofrontality in SZ: Glucose Metabolism

As typically defined, the hypofrontality of SZ refers to significant reductions in glucose metabolism and blood flow, and associated decreases in neural activity, largely confined to frontal regions of the cortex, in SZ [82]. Based on early meta-analysis of 29 functional imaging studies, Hill et al. [82] reported that the collective findings consistently demonstrated both resting state and task-based frontal hypofrontality for SZ patients. They thus concluded that “hypofrontality has become one of the most widely cited and influential findings in the literature on schizophrenia”. While effects are typically bilateral, Spironelli et al. [83] described a greater degree of hypofrontality on the left than on the right of the FrC for SZ patients, which was associated with linguistic deficits.

As noted, a reduced rate of glucose metabolism (or hypometabolism) is a major factor contributing to the hypofrontality in SZ. Accordingly, several reports have described changes in cerebral glucose utilization, primarily in the PFC, of SZ patients. In a comprehensive meta-analysis, Townsend et al. [84] examined 36 reports (1335 subjects) which assessed brain glucose metabolism in SZ, using radioactive fluorodeoxyglucose (^18^ FDG-PET). Among their main findings, they [84] described overall marked reductions in glucose metabolism of SZ patients, essentially confined to the frontal cortex—as no changes were seen in the parietal, temporal or occipital cortices. They further reported that effects were more pronounced for chronic, compared to first episode, SZ patients, suggesting a steady (or steep) decline in frontal glucose metabolism with the progression of the disease. Finally, noting that their effects were much greater than those described by Hill et al. [82] suggested that this could probably be explained by Hill et al. combining studies of glucose metabolism and blood flow, whereas they only assessed glucose metabolism—thus concluding that glucose metabolic abnormalities may be more marked than changes in blood flow in SZ [84].

### 5.3. Frontal Cortical Hypofrontality in SZ: Blood Flow

In a parallel manner to reduced PFC glucose metabolism in SZ, several studies have described significant reductions in cerebral blood flow (CBF) to the prefrontal cortex in SZ. Specifically, decreases in blood flow to the FrC/PFC have been demonstrated for first episode (neuroleptic-naïve) patients, for chronic SZ and for individuals at high risk for SZ [85,86,87,88,89,90,91,92,93].

Zhu et al. [91] examined CBF in a large population of 100 SZ patients compared to 94 healthy controls (HC) and described significant reductions in CBF in FrC regions for SZ patients. Similarly, Kindler et al. [90] demonstrated reduced blood flow to the PFC in clinically high risk (CHR) individuals, first episode SZ (FEP) and chronic SZ patients relative to controls. Interestingly, they reported [90] that reductions in CBF for chronic SZ and FEP patients were concentrated in the dorsolateral PFC, whereas decreases for CHR individuals were mainly localized to the orbitofrontal cortex. They suggested [90] that the reduced blood flow for CHR subjects likely signifies that PFC hypoperfusion is a core feature of SZ and as such, may potentially serve as a biomarker for the disease. Consistent with previous studies, Selvaggi et al. [93] demonstrated greatly reduced CBF in the frontal cortex of unmedicated FEP subjects but notably found no changes in the striatum or the hippocampus. Noting, however, that some previous studies including their own [94], described *increases* in CBF in the striatum/basal ganglia of SZ patients, they tentatively attributed this increase to the use of antipsychotics affecting the dopamine system.

To conclude, as cortical delta oscillations are associated with significant overall reductions in neuronal activity [50,51], there is correspondingly less a need to perfuse the PFC (reduced blood flow) or to utilize energy to maintain cellular activity (reduced glucose metabolism) in the ‘delta state’—thus reflecting the hypofrontality of SZ. In effect then, the PFC delta, hypofrontality and disconnection of the PFC from the thalamus in SZ are potent indicators that the PFC is disabled (or non-functional) in SZ—with obvious detrimental effects.

## 6. Neural Substrates for FrC Delta Activity, Hypofrontality and the Thalamocortical Dysconnection of SZ

As is well established, SZ entails alterations of glutamate signaling: the glutamate hypofunction model for SZ. Specifically, NMDA receptor (NMDAR) antagonists give rise to a host of positive and negative symptoms in human SZ and analogous effects in animal models of SZ [95,96,97]. Several reports in awake or anesthetized rats have shown that systemic injections of NMDAR antagonists trigger/enhance delta power in the cortex [98,99,100,101,102,103]. As the thalamus is the pacemaker for cortical EEG activity, prominently including delta [104,105], attention has focused on the thalamus as a primary site for the actions of NMDAR antagonists. For instance, Kiss and associates [101,102] demonstrated in anesthetized rats that systemic injections of MK-801 or intra-thalamic injections of lidocaine produced marked reductions in the frequency of delta oscillations in the PFC. Within the thalamus, the reticular nucleus (RT) has emerged as a principal target for the effects of NMDAR antagonists—with modulatory actions on other thalamic nuclei, namely on the nucleus reuniens (RE) [12,106,107,108,109].

A notable model [107,110] maintains that NMDAR antagonists act on the RT to initiate a cascade of effects on several interconnected structures to ultimately produce delta activity in the PFC. Specifically, as modeled, NMDAR antagonists rhythmically activate RT cells at 4 Hz which, in turn, drives RE cells and its target hippocampal neurons at delta frequency (4 Hz) to activate, through a polysynaptic pathway, dopamine (DA) neurons of the ventral tegmental area (VTA) [111,112]. DA/VTA cells project back to the thalamus to maintain heightened activity within this circuitry and to rhythmically drive TC neurons at 4 Hz, thus producing delta activity in the mPFC. Supporting this framework, Zhang et al. [111] showed that the infusion of the NMDAR antagonist, APV, into the RT in the slice preparation, rhythmically activated RT cells at delta frequency. This effect was mediated by the glutamate subunit receptor, NR2C, of RT cells [112]. RT cells containing this receptor complex become slightly depolarized to ambient glutamate and thus hyperpolarized to NMDAR antagonists which de-inactivate T-type Ca^2+^ channels to trigger delta burst activity of RT neurons. Further, the APV-induced delta bursting activity of RT cells was shown to be abolished by dopamine D2 receptor antagonists [111]. As such, Zhang et al. [111] concluded that NMDAR antagonists and DA/D2 agents work synergistically to generate delta activity in RT neurons to ultimately produce delta oscillations in the PFC during waking as “an established abnormality in schizophrenia”.

Consistent with this, several reports have demonstrated that RT cells exert delta synchronizing effects on the PFC [113,114,115,116,117,118]. For instance, Marini et al. [113] demonstrated that bilateral ibotenic acid-induced lesions of the rostral (limbic) sector of RT suppressed delta oscillations in the PFC for several days post-injection and completely eliminated delta in some rats. Cueni et al. [114] subsequently reported that the deletion of SK-type K^+^ channels in RT cells of mice abolished the oscillatory discharge of RT neurons and produced a four-fold reduction in delta activity in the cortex. In like manner, Espinosa et al. [115] showed that the deletion of the voltage-gated potassium channels, Kv3.1 and Kv3.3, which are highly expressed in RT cells, severely disrupted the discharge properties of RT neurons and resulted in a 70% reduction in cortical delta activity. Finally, several studies have shown that selectively activating RT neurons, via various methods, elicits/enhances delta wave activity in the cortex [116,117,118].

## 7. Role of the Nucleus Reuniens (RE) in a Circuitry Producing Delta Oscillations in the Hippocampus and the PFC in the Waking State—With Direct Relevance to SZ

**Figure 1:**
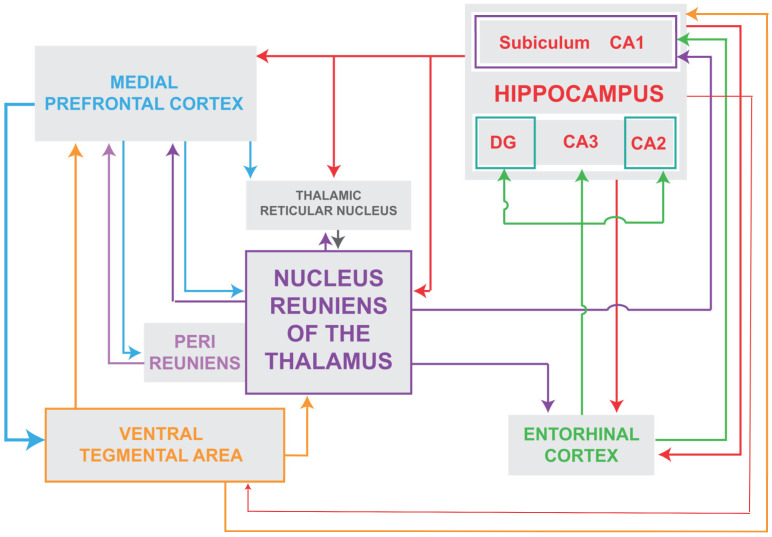
Schematic representation of the major interconnections of the nucleus reuniens of the ventral midline thalamus with subcortical and cortical structures. As depicted, the nucleus reuniens is reciprocally connected with the reticular nucleus of the thalamus, the hippocampus and the medial prefrontal cortex and receives projections from the ventral tegmental area. In addition, each of these structures are, in turn, interconnected to form loops that ultimately serve to drive delta oscillations in the prefrontal cortex. Abbreviations: DG, dentate gyrus of hippocampus; CA1, CA2, CA3, fields CA1–CA3 of Ammons horn of the hippocampus.

The nucleus reuniens (RE) of the ventral midline thalamus is a major source of afferent projections to the hippocampus (HF) and to the mPFC and is strongly reciprocally connected to both structures [43,44,45,46,47,48,119,120]. (Figure 1). Approximately 10% of RE neurons project via axon collaterals to the HF and mPFC [46,121]. While the HF projects densely to the mPFC [122], there are no direct projections from the mPFC to the hippocampus [123,124] and as such RE is the main return route from the mPFC to the HF [45]. Accordingly, RE is positioned (1) to separately or simultaneously affect the HF and the PFC; (2) to regulate the transfer of information from the mPFC to the HF; and (3) to thus coordinate the actions of the HF and mPFC in various behaviors.

As described, NMDAR antagonists rhythmically activate RT cells at delta frequency (4 Hz) which then reportedly serves to drive RE cells and its main targets at 4 Hz—to ultimately produce delta oscillations in the PFC. Supporting this, Lisman and colleagues [103,106,111] showed that systemic or intra-RE injections of the NMDAR antagonist, ketamine, produced 4 Hz activity in the RE—and importantly, via the RE, delta oscillations in CA1 of the hippocampus. Regarding the actions of RE on the HF, Zhang et al. [103] showed that following systemic ketamine injections: (1) the activity RE and HF cells became strongly correlated and highly coherent at 4 Hz; (2) ketamine-induced delta oscillations in the HF were blocked by muscimol injections in RE; and (3) the discharge of RE and HF cells became entrained to local field potentials at 4 Hz and mean firing rates of RE/HF cells increased by greater than 250%. Taken together, these findings indicate that NMDAR antagonists, acting indirectly, via RT, or directly on the RE, rhythmically drive RE neurons at delta frequency to entrain CA1 neurons, as well as those of other RE targets, including the PFC, at 4 Hz.

In addition to NMDAR hypofunction, it is well-recognized that dopamine (DA) is overexpressed in SZ (DA hyperfunction) [125]. Similar to the action of NMDAR antagonists on the RE, it appears that RE serves a direct role in the heightened release of DA in SZ. Specifically, in an initial series of studies, Grace and colleagues [125,126] described a circuit from the hippocampus to the VTA responsible for the excess release of DA in SZ. The circuit consisted of projections from the ventral subiculum (vSUB) of the HF to the nucleus accumbens, to the ventral pallidum and then to the VTA. They further demonstrated that the infralimbic cortex (IL) of the mPFC exerts a modulatory influence on the vSUB in the release of DA from the VTA; that is, the inhibition of IL triggers the release of DA from the VTA [127]. As the IL does not project to the vSUB/HF [123,124], they subsequently demonstrated that RE mediates the effects of IL on the vSUB—and then ultimately on the VTA [128,129].

Specifically, they [128] showed that (1) RE stimulation increased DA/VTA population activity; (2) this effect was blocked by the suppression of the vSUB; and (3) IL inhibition-induced excitatory actions on the VTA were abolished by suppressing RE. In a follow-up report, Zimmerman and Grace [129] demonstrated in anesthetized rats that the inhibition of IL produced a shift in RE cell discharge from (an anesthetic-like) burst pattern to tonic firing, whereas IL stimulation profoundly suppressed the activity of RE cells, resulting in the complete silencing of 75% of RE neurons. Taken together, these findings demonstrate that RE is a critical intermediary in an extended circuitry giving rise to the enhanced release of DA from the VTA.

In complimentary studies in urethane-anesthetized and awake rats, Kocsis and colleagues [130,131] described the effects of systemic administration of dopamine D4 receptor (D4R) agonists (and antagonists) on oscillatory activity of the RE, HF and mPFC—concentrating on delta (1–4 Hz) and theta oscillations. In anesthetized rats, they [130] showed that the administration of the D4R agonist, A-412997, produced marked increases in delta activity in the mPFC and corresponding decreases in theta in the HF. In a follow-up report in awake rats [131], they demonstrated that low dose injections of D4R agonists produced a ‘dramatic’ increase in delta oscillations in the RE, that is, a five-fold increase in delta power for 20 min post-injection. As with anesthetized rats [130], D4R agonists also increased delta power in the PFC. As the injections were systemic, it was not possible to determine the precise site(s) of action of D4R agents [131]. However, a likely target would appear to be the reticular nucleus (RT) of the thalamus. Specifically, the RT is among the few thalamic nuclei of rodents to receive DA input [132,133], and D4 receptors are densely concentrated in RT [134,135,136,137,138]. Additionally, the application of D4R agonists to RT in vivo has been shown to increase the activity of RT cells in a burst-like manner [137]. As previously discussed, the RT exerts delta (4 Hz) synchronizing influences on the RE and PFC, and as such RT appears ideally positioned to mediate the D4R-induced enhancement of delta oscillations of the RE and PFC [131]. Finally, the antipsychotic agent, clozapine, binds with high affinity to dopamine D4 receptors—or higher than that to the D2 receptor [139].

In summary, the RE-elicited rhythmical drive and activation of the vSUB/HF triggers (through a polysynaptic pathway) the release of DA from the VTA and DA/VTA cells project back to the thalamus to maintain (or augment) the burst discharge of RT/RE cells—an effect putatively mediated by dopamine D4 receptors of the RT. This, in turn, serves to sustain delta activity throughout the circuit and importantly to pace delta oscillations in the PFC. This circuitry is illustrated in Figure 1 showing that the RE is a hub in the interconnections of the RT, HF, PFC and VTA to ultimately produce delta oscillations in the PFC. As was previously pointed out [109], the foregoing scheme for SZ incorporates the critical elements of the disorder “such as NMDAR hypofunction, pathologic delta, increased activity in CA1 and dopaminergic hyperactivity”.

## 8. Delta Oscillations in the RE-HF-PFC Circuitry: Effects on Cognition with Relevance to SZ—A Brief Description

As is well documented, cognitive functions, including working memory, are severely impaired in SZ [27,28,29,30,31,32]. As developed herein, SZ involves a disruption to thalamo-cortical communication, and the associated abnormal presence of delta oscillations in the PFC in the waking state. The presence of delta in the PFC during waking would indicate that the PFC is disabled (or non-functional) in the waking state in SZ—thereby contributing to the cognitive deficits of SZ.

As described (see above), the RE is a major source (or trigger) for the elicitation of delta activity in the PFC and the HF. Further, as well established, RE serves a critical role in memory processing functions, prominently including working memory. Specifically, the inactivation of RE has been shown to ‘severely’ disrupt mnemonic functions of the HF and PFC (for review, [47,48,119,140,141,142,143,144]). Accordingly, the RE-induced delta oscillations of the PFC would severely disrupt the normal activity/functions of the PFC which, in turn, would produce several PFC-associated deficits in SZ, including impairments of memory/working memory. In this regard, Daun et al. [145] demonstrated that optogenetic stimulation of RE-hippocampal projecting fibers at delta frequencies disrupted working memory (WM) in rats. They suggested that the WM deficits may result from delta interfering with (or blocking) normal oscillations (theta, gamma) of the HF responsible for proper hippocampal functioning. In this regard, it is noteworthy that in addition to robustly increasing delta activity in the RE, D4R agonists were shown to significantly suppress theta in the hippocampus [131].

## 9. Conclusions

Schizophrenia (SZ) is a severe mental disorder associated with an array of symptoms characterized as positive, negative and cognitive dysfunctions. While SZ is a multifaceted disorder affecting several regions of the brain, alterations of thalamocortical systems have emerged as a leading contributor to SZ. Specifically, it has been shown that (1) the thalamus is functionally disconnected from the prefrontal cortex (PFC) in SZ; (2) neural activity and blood flow to the PFC are greatly diminished in SZ (hypofrontality); and (3) delta oscillations are abnormally present in the PFC during waking in SZ. Each of these signs indicate a major disruption of the normal activity/function of the PFC. We suggest that these events are triggered by abnormal delta activity during waking. Specifically, the decreased neural activity during delta, and associated reduced energy demands/utilization with delta, would further suppress PFC activity leading to the hypofrontality of SZ. In addition, abnormal delta oscillations, coupled with reduced PFC activity, may contribute to the mismatch between thalamic and cortical activity in SZ—or the functional TC dysconnectivity of SZ. In sum, the abnormal delta, hypofrontality and TC dysconnection of SZ are potent indicators that the prefrontal cortex is disabled (or non-functional) in SZ.

A circuitry was described that produces delta oscillations in the PFC in SZ. Specifically, NMDAR antagonists rhythmically activate cells of the reticular nucleus of the thalamus at 4 Hz which, in turn, drives cells of the nucleus reuniens (RE) and its target hippocampal neurons at delta frequency (4 Hz) to activate, through a polysynaptic pathway, dopamine (DA) neurons of the ventral tegmental area (VTA). DA/VTA cells project back to the thalamus to maintain heightened activity within this circuitry and to rhythmically drive TC neurons at 4 Hz, thus producing delta activity in the PFC. As SZ involves reduced glutamate signaling (NMDAR hypofunction) and enhanced dopamine signaling (DA hyperfunction), it has been shown that both NMDAR antagonists and dopamine agonists produce pronounced increases in delta oscillations in nucleus reuniens of the midline thalamus. This would indicate that the nucleus reuniens is a primary source for the initiation of delta activity in the PFC in SZ. As discussed, the abnormal PFC delta oscillations during waking would signify that the PFC is essentially non-functional in SZ—thus contributing to various deficits of SZ, including impairments of memory.

## Data Availability

No new data were created or analyzed in this study. Data sharing is not applicable to this article.
